# First-line pembrolizumab for advanced and metastatic non-small cell lung cancer with a PD-L1 TPS ≥ 50% at a Portuguese centre

**DOI:** 10.36416/1806-3756/e20250231

**Published:** 2026-03-05

**Authors:** Maria João Cavaco, Direndra Hasmucrai, Filipa Ferro, Andrea Lopes Machado, Ana Sofia Vilariça, Paula Alves

**Affiliations:** 1. Serviço de Pneumologia, Unidade Local de Saúde Oeste, Torres Vedras, Portugal.; 2. Serviço de Pneumologia, Unidade Local de Saúde Santa Maria, Lisboa, Portugal.

**Keywords:** Antibodies, monoclonal, humanized, Carcinoma, non-small-cell lung, Biomarkers, tumor, Programmed cell death 1 receptor, B7-H1 antigen

## Abstract

**Objective:**

Pembrolizumab monotherapy is an established first-line treatment option in patients with locally advanced or metastatic non-small cell lung cancer (NSCLC) expressing programmed death ligand 1 (PD-L1) with a tumour proportion score (TPS) ≥ 50%. However, real-life data are scarce. The aim of this study was to evaluate the effectiveness and safety of pembrolizumab in such patients and to identify predictive factors for progression-free survival (PFS) and overall survival (OS).

**Methods:**

This was a retrospective observational study of patients with advanced/metastatic NSCLC, expressing PD-L1 with a TPS ≥ 50%, treated with first-line pembrolizumab monotherapy at a Portuguese centre between January of 2017 and December of 2023. We assessed PFS, OS and safety.

**Results:**

Of the 101 patients included, 67.3% were men, 92.1% were White, 89.1% had a performance status 0-1, 76.2% had a non-squamous histology and 90.1% had metastatic disease. The median age was 65 years. The median PFS and OS were 11.0 months (95% CI: 7.03-14.97) and 15.0 months (95% CI: 10.81-19.19), respectively. Immune-mediated adverse events occurred in 31.7% of the cases. Multivariate analysis identified squamous histology as a negative predictive factor for PFS (HR = 2.60; 95% CI: 1.48-4.50; p = 0.001) and OS (HR = 2.28; 95% CI: 1.30-3.97; p = 0.004) and showed brain metastases to be associated with shorter OS (HR = 1.98; 95% CI: 0.90-4.36; p = 0.026).

**Conclusions:**

This study supports the effectiveness and safety of first-line pembrolizumab monotherapy in patients with advanced/metastatic NSCLC expressing PD-L1 with a TPS ≥ 50%, highlighting the disparity of outcomes according to histology.

## INTRODUCTION

Lung cancer is the leading cause of cancer-related death worldwide.^1^ In Portugal, more than 5,000 patients are diagnosed with non-small cell lung cancer (NSCLC) each year.^1^ Nearly half of these patients are diagnosed at an advanced stage, and, even when NSCLC is detected at an early stage, a significant number of patients will eventually progress to metastatic disease.^1^ Historically, these patients have a poor prognosis despite the use of platinum-based chemotherapy. Over the past decade, the treatment of NSCLC without known actionable gene mutations has undergone a major paradigm shift with the development of immune checkpoint inhibitors (ICIs).^2^

Pembrolizumab, a humanised monoclonal antibody against programmed death 1, has been shown to improve overall survival (OS), progression-free survival (PFS) and the objective response rate (ORR) in locally advanced and metastatic NSCLC, especially in the subset of patients expressing programmed death ligand 1 (PD-L1) with a tumour proportion score (TPS) ≥ 50%.^3^ ^,^ ^4^ The phase III randomised controlled trials (RCTs) designated KEYNOTE-024 and KEYNOTE-042 established pembrolizumab monotherapy as a first-line treatment option in these patients, with an overall good safety profile.^3^ ^,^ ^4^

The updated long-term follow-up data of KEYNOTE-024 showed a median PFS of 7.7 months (95% CI: 6.1-10.2 months) in the group treated with pembrolizumab, compared with 5.5 months (95% CI: 4.2-6.2 months) in the chemotherapy group, and improved overall survival (OS) of 26.3 months (95% CI: 18.3-40.4 months) for the pembrolizumab group, compared with 13.4 months (95% CI: 9.4-18.3 months) for the chemotherapy group. In patients with a PD-L1 TPS ≥ 50%, five-year PFS and OS rates after pembrolizumab treatment have been estimated at 12.8% and 31.9%, respectively.^5^ Similar results were found in the subgroup of NSCLC patients with a PD-L1 TPS ≥ 50% who were treated with pembrolizumab monotherapy in the KEYNOTE-042 RCT, in which the reported median PFS and OS rates were 6.5 months (95% CI: 5.9-8.6) and 20.0 months (95% CI: 15.9-24.2), respectively.^6^ Five-year estimates were slightly lower in that study: 9.2% for PFS and 21.9% for OS.^6^ The ORR was 46.1% in KEYNOTE-024 and 39.1% in KEYNOTE-042.^5^ ^,^ ^6^ Regarding safety, the incidence of immune-mediated adverse events (AEs) in KEYNOTE-024 and KEYNOTE-042 was 34.4% (grade 3-5 in 13.6%) and 27.5%, respectively.^5^ ^,^ ^6^

There are few available real-life data on the effect of first-line pembrolizumab in patients with advanced NSCLC expressing PD-L1 with a TPS ≥ 50%. As the inclusion criteria for these RCTs are quite restrictive, patients included in these trials may differ from those treated in a real-life setting. Most real-life studies performed to date have been retrospective observational studies with follow-up times shorter than those of the RCTs. Nevertheless, the median real-life PFS and OS rates reported have ranged from 8.2 to 10.6 months and from 12.6 to 25.5 months, respectively.^7^ ^-^ ^13^

Predicting who will respond to pembrolizumab monotherapy remains a challenge. Various baseline factors related to not only the patient-such as age, sex, performance status (PS) and smoking history- but also the tumour-including histology, mutations and number/location of metastases-have been investigated.^9^ ^-^ ^15^ Retrospective studies have identified several possible independent negative predictive factors for PFS and OS, including non-smoker status, a PS ≥ 2, a neutrophil/lymphocyte ratio > 4, baseline tumour size, squamous histology, presence of brain, bone or liver metastases and ≥ 3 metastatic sites.^9^ ^-^ ^15^ However, results from these exploratory unadjusted analyses should be considered with caution. 

The aim of this study was to investigate the real-life effectiveness and safety of pembrolizumab in patients with advanced NSCLC expressing PD-L1 with a TPS ≥ 50% at a Portuguese centre, as well as to identify potential predictive factors for PFS and OS.

## METHODS

This was a non-interventional, retrospective study conducted in the Pulmonary Oncology Department of the Santa Maria Hospital, in the city of Lisbon, Portugal. We reviewed the records for all consecutive eligible patients that received at least one dose of pembrolizumab monotherapy as a first-line treatment for advanced localised or metastatic NSCLC expressing PD-L1 with a TPS ≥ 50% between January of 2017 and December of 2023. The selected patients were followed until December of 2024. 

Patient data were obtained from medical records, including demographics and baseline clinical features, as well as tumour- and treatment-related data. Tumour response was evaluated according to the Response Evaluation Criteria in Solid Tumours guideline, version 1.1.^16^ Information regarding subjects was managed with anonymous numerical codes. All procedures were conducted in accordance with the Declaration of Helsinki and in compliance with national regulations.

The primary endpoints were PFS, defined as the time from treatment initiation to the date of radiologic disease progression or death, and OS, defined as the time from treatment onset to death from any cause. Secondary endpoints included the ORR (the proportion of patients with complete or partial response), the disease control rate (the proportion of patients with an ORR or stable disease) and safety. All AEs were graded according to the Common Terminology Criteria for Adverse Events, version 5.0.^17^

Statistical analysis was performed with the IBM SPSS Statistics software package, version 29.0 (IBM Corp., Armonk, NY, USA). A p-value below 0.05 was considered statistically significant. Descriptive analyses were used to characterise the most relevant clinical variables. Variables are presented as medians for continuous variables and as percentages for categorical variables. The median follow-up period was calculated with the reverse Kaplan-Meier method, whereas the PFS and OS rates were calculated with the standard Kaplan-Meier method. Univariate Cox regression analyses were used in order to estimate hazard ratios (HRs) and 95% confidence intervals. Variables that showed a significant effect were included in multivariate Cox regression analyses, in order to identify independent prognostic factors.

## RESULTS

Of the 101 patients included (Table 1), 68 (67.3%) were men, 93 (92.1%) were White and 68 (67.3%) had a PS of 1. The median age was 65 years (range, 44-95 years). Twenty-four patients (23.8%) had a tumour with squamous histology, and 36 (35.6%) had oncogene-addicted NSCLC, without first-line targeted treatment available at the time of pembrolizumab initiation. Fourteen patients (13.9%) had tumours that were detected at an early stage but progressed to advanced or metastatic disease. Ninety-one patients (90.1%) had metastatic disease, and 53 (52.5%) of those patients ≥ 3 metastases. Brain metastases were present in 24 patients (23.8%), of whom 10 (9.9%) underwent brain radiotherapy and 7 (6.9%) were treated with surgery. 

**Table 1:** Baseline demographic and clinical characteristics of patients with advanced non-small-cell lung cancer (N = 101).

Characteristic	n (%)	Characteristic	n (%)
Age (years)		Histology	
< 65	39 (38.6)	Non-squamous	77 (76.2)
≥ 65	62 (61.4)	Squamous	24 (23.8)
Median	65	Mutations	
Range	44-95	*KRAS*	27 (26.7)
Sex		*BRAF*	3 (2.9)
Male	68 (67.3)	*EGFR*	2 (2.0)
Female	33 (32.7)	*MET*	2 (2.0)
Race		*NRAS*	1 (1.0)
White	92 (91.1)	*RET*	1 (1.0)
Non-White	9 (8.9)	Number of metastases	
Performance status		< 2	48 (47.5)
0	22 (21.8)	≥ 3	53 (52.5)
1	68 (67.3)	Location of metastases	
≥ 2	11 (10.9)	≥ 3 organs or systems	30 (29.7)
Smoking status		Bone	44 (43.6)
Never	6 (5.9)	Brain	24 (23.8)
Current	57 (56.5)	Adrenal gland	20 (19.8)
Former	38 (37.6)	Liver	9 (8.9)
Comorbidities		Brain treatment	
COPD	27 (26.7)	Radiotherapy	10 (9.9)
Cardiovascular disease	19 (18.8)	Surgery	7 (6.9)
Diabetes mellitus	18 (17.8)	Palliative radiotherapy	
History of other tumours	13 (12.9)	Bone	23 (22.8)
Stage		Previous systemic therapy	
Locally advanced (III)	9 (8.9)	Any	14 (13.9)
Metastatic (IV)	91 (90.1)	Chemoradiotherapy	8 (7.9)
IVA	38 (37.2)	Adjuvant chemotherapy	4 (4.0)
IVB	53 (52.0)	Neoadjuvant chemotherapy	2 (2.9)

**Figure 1:**
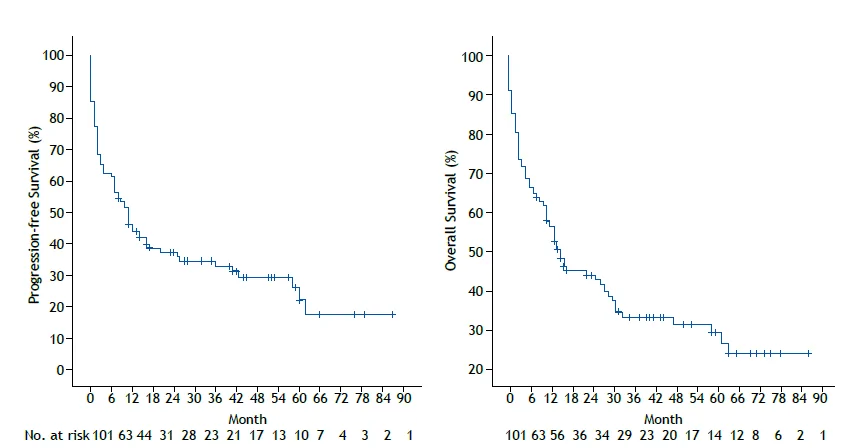
Kaplan-Meier curves of progression-free and overall survival.

At the time of data cut-off, with a median follow-up of 23 months (95% CI: 37.70-50.30 months), 75 (74.2%) of the patients had progressed or died (Figure 1). The median PFS was 11.0 months (95% CI: 7.03-14.97 months) and the median OS was 15.0 months (95% CI: 10.81-19.19 months). The Kaplan-Meier estimates were 37% and 43% for two-year PFS and OS, respectively, whereas they were 26% and 28% for five-year PFS and OS, respectively.

Of the 101 patients evaluated, 69 (68.3%) completed at least 3 cycles of pembrolizumab. At data cut-off, 15 patients were still receiving treatment. The ORR was 44.5%, and the disease control rate was 66.3%. Five patients had a complete response. Twenty-one patients (20.8%) completed ≥ 35 cycles of pembrolizumab, with an ORR of 95.2% in this subgroup. Baseline characteristics of these patients were similar to those of the total study population.

Univariate Cox regressions (Table 2) showed the following factors to be associated with shorter PFS: non-White race (HR = 2.19; 95% CI: 1.04-4.62; p = 0.040), squamous histology (HR = 2.51; 95% CI: 1.49-4.22; p = 0.001), ≥ 3 metastases (HR = 1.73; 95% CI: 1.07-2.77; p = 0.025) and the need for brain radiotherapy (HR = 2.06; 95% CI: 1.01-4.17; p = 0.045). In the multivariate analysis (Table 3), only squamous histology retained significance as an independent predictive factor for PFS (HR = 2.60; 95% CI: 1.48-4.50; p = 0.001).

**Table 2:** Univariate Cox regressions for progression-free and overall survival.

Characteristic	Progression-free survival			Overall survival		
	HR	95% CI	p	HR	95% CI	p
Age ≥ 65 years	1.31	0.79-2.15	0.296	1.32	0.80-2.18	0.281
Male sex	0.76	0.45-1.28	0.310	0.71	0.42-1.21	0.206
White race	2.19	1.04-4.62	0.040	2.62	1.24-5.56	0.012
ECOG PS ≥ 1 ≥ 2	1.18 1.53	0.65-2.13 0.76-3.09	0.581 0.237	1.54 1.64	0.84-2.85 0.84-3.22	0.167 0.152
Smoking status Never Current	0.55 0.73	0.24-1.29 0.46-1.18	0.168 0.200	0.61 0.77	0.26-1.41 0.47-1.23	0.246 0.273
Comorbidities COPD Cardiovascular disease Diabetes mellitus History of other tumours	0.69 0.89 1.56 2.10	0.40-1.20 0.44-1.81 0.77-3.17 0.87-5.03	0.189 0.753 0.216 0.098	0.65 1.42 1.12 1.91	0.36-1.15 0.79-2.56 0.94-1.31 0.99-3.69	0.138 0.244 0.199 0.054
Squamous histology	2.51	1.49-4.22	0.001	2.43	1.43-4.10	0.001
Mutations Presence *KRAS*	1.33 0.82	0.77-2.29 0.47-1.44	0.308 0.499	1.46 0.88	0.84-2.54 0.50-1.57	0.178 0.668
Stage IV	1.18	0.54-2.58	0.638	1.27	0.55-2.94	0.571
Metastases ≥ 3 ≥ 3 organs or systems Bone Brain Adrenal gland Liver	1.73 0.86 1.47 1.65 1.24 1.36	1.07-2.77 0.51-1.43 0.92-2.35 0.97-2.81 0.67-2.29 0.62-2.98	0.025 0.557 0.109 0.063 0.500 0.443	1.57 0.94 1.48 1.71 0.76 1.28	0.97-2.55 0.56-1.59 0.92-2.38 1.01-2.92 0.41-1.42 0.58-2.80	0.067 0.835 0.110 0.048 0.394 0.543
Treatment for brain metastases Radiotherapy Surgery	2.06 0.86	1.01-4.17 0.31-2.36	0.045 0.767	1.94 1.05	0.92-4.08 0.42-2.61	0.082 0.920
Palliative radiotherapy	1.19	0.70-2.04	0.521	1.17	0.68-2.04	0.565
Previous systemic therapy Any Chemoradiotherapy Adjuvant chemotherapy Neoadjuvant chemotherapy	0.86 0.81 0.79 1.26	0.44-1.70 0.35-1.90 0.19-3.27 0.30-5.22	0.668 0.634 0.749 0.749	0.72 0.65 0.86 0.49	0.34-1.51 0.23-1.78 0.21-3.53 0.07-3.50	0.380 0.396 0.836 0.473

HR: hazard ratio; and PS: performance status.

**Table 3:** Multivariate Cox regressions for progression-free and overall survival.

Characteristic	Progression-free survival			Overall survival		
	HR	95% CI	p	HR	95% CI	p
Non-White race	1.56	0.71-3.42	0.274	1.98	0.90-4.36	0.089
Squamous histology	2.60	1.48-4.50	0.001	2.28	1.30-3.97	0.004
≥ 3 metastases	1.60	0.95-2.57	0.069			
Brain metastases				1.98	0.90-4.36	0.026
Brain radiotherapy	2.00	0.94-4.29	0.074			

HR: hazard ratio.

In the univariate Cox regression, the following factors were shown to be associated with shorter OS: non-White race (HR = 2.62; 95% CI: 1.24-5.56; p = 0.012), squamous histology (HR = 2.43; 95% CI: 1.43-4.10; p = 0.001) and the presence of brain metastases (HR = 1.71; 95% CI: 1.01-2.92; p = 0.048). In the multivariate analysis, the factors that retained significance were squamous histology (HR = 2.28; 95% CI: 1.30-3.97; p = 0.004) and the presence of brain metastases (HR = 1.98; 95% CI: 0.90-4.36; p = 0.026).

**Figure 2:**
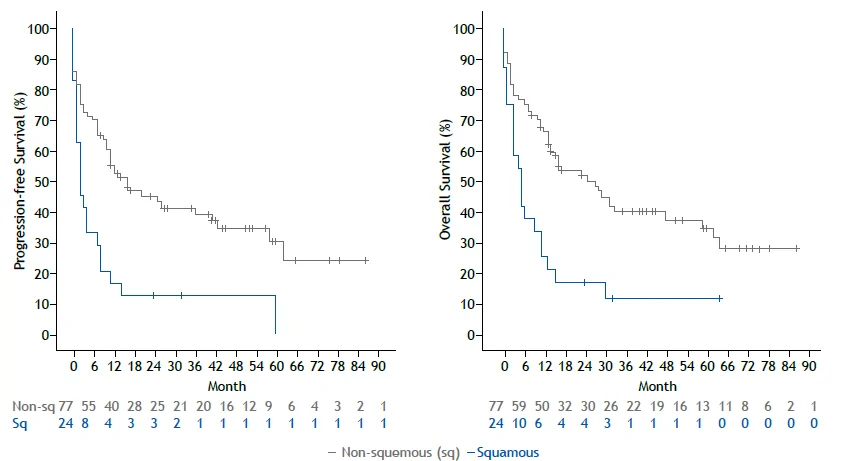
Kaplan-Meier curves of survival probability according to histology. sq: squamous.

The most relevant predictive factor for PFS and OS was the histology (Figure 2). For patients with non-squamous NSCLC, the median PFS and OS were 16 months (95% CI: 2.98-29.02 months) and 25 months (95% CI: 12.57-37.43 months), respectively, significantly longer than the 2 months (95% CI: 0.09-3.91 months) and 5 months (95% CI: 2.63-7.37 months), respectively, observed for patients with squamous NSCLC.

Regarding safety, immune-mediated AEs occurred in 32 patients (31.7%), including 14 (13.9%) who experienced AEs categorized as grade 3 or 4. The most common AEs were hypothyroidism, seen in 6 patients (5.9%); skin reaction, also seen in 6 (5.9%); pneumonitis, seen in 4 (4.0%); hypophysitis, also seen in 4 (4.0%); and nephritis, seen in 3 (3.0%). Six patients (5.9%) had to discontinue treatment: 2 (4.0%) because of hypophysitis; 1 (1.0%) because of colitis; 1 (1.0%) because of pneumonitis; 1 (1.0%) because of nephritis; and 1 (1.0%) because of a severe skin reaction. There were no treatment-related deaths.

## DISCUSSION

To our knowledge, this is the first real-life study on the effectiveness and safety of first-line pembrolizumab for advanced NSCLC with a PD-L1 TPS ≥ 50% at a Portuguese centre. Baseline demographic characteristics were similar to those reported in KEYNOTE-024 and KEYNOTE-042,^5^ ^,^ ^6^ except for the inclusion of patients with a PS ≥ 2 (10.9%) and a predominance of White patients. Regarding disease characteristics, it is interesting to note that our study population included a higher proportion of patients with brain metastases (23.8%), as well as those including patients with oncogene-addicted NSCLC (35.6%) and patients with locally advanced disease (8.9%).

Our findings that the real-life PFS was 11.0 months and that the real-life OS was 15.0 months are consistent with those of other real-life observational studies.^7^ ^-^ ^13^ The PFS in our study population was superior to the 7.7 months reported in KEYNOTE-024 and the 6.5 months reported for the subgroup of NSCLC with a PD-L1 TPS ≥ 50% in KEYNOTE-042, whereas the OS in our study population was inferior to the 26.0 and 20.0 months reported in those two RCTs, respectively.^5^ ^,^ ^6^ The ORR and AE rate observed in the present study were also similar to those reported in those pivotal RCTs and in other retrospective real-life studies.

In the present study, squamous histology was predictive of worse outcomes (PFS and OS). This is in line with several reports showing survival to be shorter for squamous NSCLC than for non-squamous NSCLC.^18^ However, to our knowledge, median PFS and OS values by histological type from the RCTs have not been published to date. We believe that difference in the five-year survival rates between KEYNOTE-024 and KEYNOTE-042 (31.9% vs. 21.9%) could be at least partially due to the difference in the proportion of patients with squamous histology enrolled in those studies (18.8% vs. 38.1%).^5^ ^,^ ^6^ Nonetheless, it is important to note that this outcome has not been consistently found in all of the real-life retrospective studies.^12^ ^,^ ^13^

Concerning the number and location of metastases, the presence of brain metastases led to shorter OS in the present study. Of the patients in our study population, 23.8% had brain metastases at diagnosis, which is notably higher than the 5.5% reported in KEYNOTE-042 but concordant with previous retrospective studies.^6^ ^,^ ^7^ ^,^ ^9^ ^,^ ^11^ ^,^ ^12^ In addition, 16.8% of our patients needed treatment for brain metastases, compared with 11.7% of those in KEYNOTE-042.^5^ Little is known about ICI activity in the central nervous system. A recent meta-analysis reported that the treatment effect of ICI versus chemotherapy is smaller in patients with brain metastases than in those without.^19^ It is also important to note that patients with brain metastases more frequently received treatment with corticosteroids, which has been shown to reduce the OS and PFS of patients with NSCLC under ICI therapy.^21^ ^,^ ^22^ We were unable to evaluate corticosteroid use in the present study. Regarding treatment for brain metastases in our study population, radiotherapy was associated with worse PFS, although only in the univariate analysis. Nannini et al.^20^ reported surgery for brain metastases before ICI therapy as the only factor significantly associated with improved PFS and OS in the multivariate analysis. More research is needed in order to understand the need for and benefits of a multimodal approach, including local and systemic treatments, in patients with brain metastases.

Another noteworthy finding of the present study is that the presence of ≥ 3 metastases, regardless of the number of organs or systems affected, was associated with worse PFS in the univariate analysis. This variable was included as a possible practical surrogate marker for tumour burden. Several studies have reported tumour burden to be linked to the effectiveness of ICI. In previous retrospective studies, a cut-off of ≥ 3 metastatic sites was frequently used.^10^ ^,^ ^23^ ^-^ ^26^ Some of these studies found that having ≥ 3 metastatic organs or systems is a risk factor for hyperprogressive disease or rapid progression.^24^ ^-^ ^26^ Tambo et al.^10^ reported that the presence of ≥ 3 metastatic sites was associated with shorter PFS and OS in the multivariate analysis.^10^ Multiple studies have also explored the relationship between baseline tumour size and survival.^14^ ^,^ ^23^ While baseline tumour size is considered to be an accurate way to assess tumour burden, it is not easily applicable in day-to-day clinical practice. More studies are needed in order to fully determine whether any number or location of metastases is an accurate surrogate marker for tumour burden. 

Our study population included a relatively low proportion of non-White patients (8.9%), mainly Black patients (5.9%), with baseline characteristics similar to those of the total study population. This variable was associated with shorter OS and PFS only in the univariate analysis. However, because of the small number of patients included, this result may not be representative. The populations evaluated in KEYNOTE-024 and KEYNOTE-042 were more diverse, including patients from different regions, such as East Asia, the European Union and Latin America.^5^ ^,^ ^6^ Very few studies have evaluated the impact of race on the survival outcomes of advanced NSCLC, and, to our knowledge, none in the subgroup of those with a PD-L1 TPS ≥ 50%. Although some studies have indicated that there are no significant racial differences in treatment effectiveness,^28^ ^-^ ^30^ others suggest that there could be differences due to longer time to diagnosis or socioeconomic factors.^30^ This result must be viewed with caution, and further research is needed in order to understand these dynamics and to develop strategies to address any disparities.

Our results show that PS, sex and smoking status, which are some of the most commonly identified prognostic factors,^9^ ^,^ ^11^ ^-^ ^13^ ^,^ ^15^ did not have an independent impact on PFS or OS. Regarding PS and smoking status, the lack of an impact might be due to the small number of never-smokers (5.9%) and patients with a PS ≥ 2 (10.9%) in our sample, implying insufficient power to fully assess the impact on survival. It may also be important to consider the factors contributing to poor PS, as studies have shown that patients starting pembrolizumab with a PS = 2 due to comorbidities tend to have a significantly better prognosis than those with a poor PS caused by tumour-related symptoms.^13^ In the present study, we were not able to differentiate between these two causes of poor PS.

The present study has several limitations. First, although patients were consecutively enrolled, it was a retrospective study comparing outcomes between non-randomised patients, making it prone to selection bias. Second, retrospective studies can be affected by data recording errors and incomplete information. For instance, data on AEs may be insufficient, with potentially underreported grade 1 and 2 AEs. However, the study benefited from a long median follow-up time and a low number of censored cases within the follow-up period, as well as multivariate analysis of various patient- and disease-related variables, leading to a comprehensive investigation regarding potential predictive factors for PFS and OS. 

In conclusion, the findings of this study support the effectiveness and safety of first-line pembrolizumab monotherapy in patients with advanced NSCLC expressing PD-L1 with a TPS ≥ 50%. They also provide an overview of the possible patient and disease characteristics that should be considered when prescribing pembrolizumab monotherapy in these patients, demonstrating that PFS and OS were shorter in patients with squamous cell carcinoma. Although observational studies offer valuable insights into real-life data, there is a need for further investigations, preferably large prospective studies, to identify clinical predictors of long-term survival in patients with advanced NSCLC and a PD-L1 TPS ≥ 50% treated with pembrolizumab.

## References

[1] World Health Organization. International Agency for Research on Cancer (IARC) (c2024). Lung Cancer Statistics: Globocan 2022.

[2] Hendriks LE, Kerr KM, Menis J, Mok TS, Nestle U, Passaro A (2023). Non-oncogene-addicted metastatic non-small-cell lung cancer ESMO Clinical Practice Guideline for diagnosis, treatment and follow-up. Ann Oncol.

[3] Reck M, Rodríguez-Abreu D, Robinson AG, Hui R, Csoszi T, Fülöp A (2016). Pembrolizumab versus Chemotherapy for PD-L1-Positive Non-Small-Cell Lung Cancer. N Engl J Med.

[4] Mok TSK, Wu YL, Kudaba I, Kowalski DM, Cho BC, Turna HZ (2019). Pembrolizumab versus chemotherapy for previously untreated, PD-L1-expressing, locally advanced or metastatic non-small-cell lung cancer (KEYNOTE-042) a randomised, open-label, controlled, phase 3 trial. Lancet.

[5] Reck M, Rodríguez-Abreu D, Robinson AG, Hui R, Csoszi T, Fülöp A (2021). Five-Year Outcomes With Pembrolizumab Versus Chemotherapy for Metastatic Non-Small-Cell Lung Cancer With PD-L1 Tumor Proportion Score = 50. J Clin Oncol.

[6] de Castro G, Kudaba I, Wu YL, Lopes G, Kowalski DM, Turna HZ (2023). Five-Year Outcomes With Pembrolizumab Versus Chemotherapy as First-Line Therapy in Patients With Non-Small-Cell Lung Cancer and Programmed Death Ligand-1 Tumor Proportion Score = 1% in the KEYNOTE-042 Study. J Clin Oncol.

[7] Amrane K, Geier M, Corre R, Léna H, Léveiller G, Gadby F (2020). First-line pembrolizumab for non-small cell lung cancer patients with PD-L1 =50% in a multicenter real-life cohort The PEMBREIZH study. Cancer Med.

[8] Bérard G, Guévremont C, Marcotte N, Schroeder C, Bouchard N, Rajan R (2023). Descriptive Analysis of First-Line Non-Small Cell Lung Cancer Treatment with Pembrolizumab in Tumors Expressing PD-L1 = 50% in Patients Treated in Quebec's University Teaching Hospitals (DALP-First Study). Curr Oncol.

[9] Decroisette C, Greillier L, Curcio H, Pérol M, Ricordel C, Auliac JB (2023). Three-Year Overall Survival of Patients With Advanced Non-Small-Cell Lung Cancers With =50% PD-L1 Expression Treated With First-Line Pembrolizumab Monotherapy in a Real-World Setting (ESCKEYP GFPC Study). J Immunother.

[10] Tambo Y, Sone T, Nishi K, Shibata K, Kita T, Araya T (2025). Five-year efficacy and safety of pembrolizumab as first-line treatment in patients with non-small cell lung cancer with PD-L1 tumor proportion score =50 % A multicenter observational study. Lung Cancer.

[11] Pons-Tostivint E, Hulo P, Guardiolle V, Bodot L, Rabeau A, Porte M (2023). Real-world multicentre cohort of first-line pembrolizumab alone or in combination with platinum-based chemotherapy in non-small cell lung cancer PD-L1 = 50. Cancer Immunol Immunother.

[12] Cortellini A, Tiseo M, Banna GL, Cappuzzo F, Aerts JGJV, Barbieri F (2020). Clinicopathologic correlates of first-line pembrolizumab effectiveness in patients with advanced NSCLC and a PD-L1 expression of = 50% Cancer Immunol. Immunother.

[13] Facchinetti F, Mazzaschi G, Barbieri F, Passiglia F, Mazzoni F, Berardi R (2020). First-line pembrolizumab in advanced non-small cell lung cancer patients with poor performance status. Eur J Cancer.

[14] Bureau M, Chatellier T, Perennec T, Goronflot T, Greilsamer C, Chene AL (2022). Baseline tumour size is an independent prognostic factor for overall survival in PD-L1 = 50% non-small cell lung cancer patients treated with first-line pembrolizumab. Cancer Immunol Immunother.

[15] Cafaro A, Foca F, Nanni O, Chiumente M, Coppola M, Russi A (2024). Real-World Safety and Outcome of First-Line Pembrolizumab Monotherapy for Metastatic NSCLC with PDL-1 Expression = 50% A National Italian Multicentric Cohort ("PEMBROREAL" Study). Cancers (Basel).

[16] Eisenhauer EA, Therasse P, Bogaerts J, Schwartz LH, Sargent D, Ford R (2009). New Response Evaluation Criteria in Solid Tumours Revised RECIST Guideline (Version 1.1). Eur J Cancer.

[17] U.S. Department of Health and Human Services (2017). National Cancer Institute. Common Terminology Criteria for Adverse Events (CTCAE) Version 5.0.

[18] Socinski MA, Obasaju C, Gandara D, Hirsch FR, Bonomi P, Bunn PA (2018). Current and Emergent Therapy Options for Advanced Squamous Cell Lung Cancer. J Thorac Oncol.

[19] Wang Y, Zhang Q, Chen C, Hu Y, Miao L, Zhou Y (2021). Association of Brain Metastases With Immune Checkpoint Inhibitors Efficacy in Advanced Lung Cancer A Systematic Review and Meta-Analysis. Front Oncol.

[20] Nannini S, Guisier F, Curcio H, Ricordel C, Demontrond P, Abdallahoui S (2024). Outcomes of Patients with Non-Small Cell Lung Cancer and Brain Metastases Treated with the Upfront Single Agent Pembrolizumab A Retrospective and Multicentric Study of the ESCKEYP GFPC Cohort. Curr Oncol.

[21] Zhang H, Li X, Huang X, Li J, Ma H, Zeng R (2021). Impact of corticosteroid use on outcomes of non-small-cell lung cancer patients treated with immune checkpoint inhibitors A systematic review and meta-analysis. J Clin Pharm Ther.

[22] Li N, Zheng X, Gan J, Zhuo T, Li X, Yang C (2023). Effects of glucocorticoid use on survival of advanced non-small-cell lung cancer patients treated with immune checkpoint inhibitors. Chin Med J (Engl).

[23] Katsurada M, Nagano T, Tachihara M, Kiriu T, Furukawa K, Koyama K (2019). Baseline Tumor Size as a Predictive and Prognostic Factor of Immune Checkpoint Inhibitor Therapy for Non-small Cell Lung Cancer. Anticancer Res.

[24] Chen Y, Hu J, Bu F, Zhang H, Fei K, Zhang P (2020). Clinical characteristics of hyperprogressive disease in NSCLC after treatment with immune checkpoint inhibitor a systematic review and meta-analysis. BMC Cancer.

[25] Zhang L, Bai L, Liu X, Liu Y, Li S, Liu J (2020). Factors related to rapid progression of non-small cell lung cancer in Chinese patients treated using single-agent immune checkpoint inhibitor treatment. Thorac Cancer.

[26] Ferrara R, Mezquita L, Texier M, Lahmar J, Audigier-Valette C, Tessonnier L (2018). Hyperprogressive Disease in Patients With Advanced Non-Small Cell Lung Cancer Treated With PD-1/PD-L1 Inhibitors or With Single-Agent Chemotherapy. JAMA Oncol.

[27] Sakata Y, Kawamura K, Ichikado K, Shingu N, Yasuda Y, Eguchi Y (2019). Comparisons between tumor burden and other prognostic factors that influence survival of patients with non-small cell lung cancer treated with immune checkpoint inhibitors. Thorac Cancer.

[28] Peravali M, Ahn J, Chen K, Rao S, Veytsman I, Liu SV (2021). Safety and Efficacy of First-Line Pembrolizumab in Black Patients with Metastatic Non-Small Cell Lung Cancer. Oncologist.

[29] Pasli M, Kannaiyan R, Namireddy P, Walker P, Muzaffar M (2023). Impact of Race on Outcomes of Advanced Stage Non-Small Cell Lung Cancer Patients Receiving Immunotherapy. Curr Oncol.

[30] Uprety D, Seaton R, Hadid T, Mamdani H, Sukari A, Ruterbusch JJ (2024). Racial and socioeconomic disparities in survival among patients with metastatic non-small cell lung cancer. J Natl Cancer Inst.

